# Supplementary figures and images for: Understanding mental health trends during COVID-19 pandemic in the United States using network analysis
Source: PLoS One. 2023 Jun 8;18(6):e0286857. doi: 10.1371/journal.pone.0286857 (PMC10249855; doi:10.1371/journal.pone.0286857)

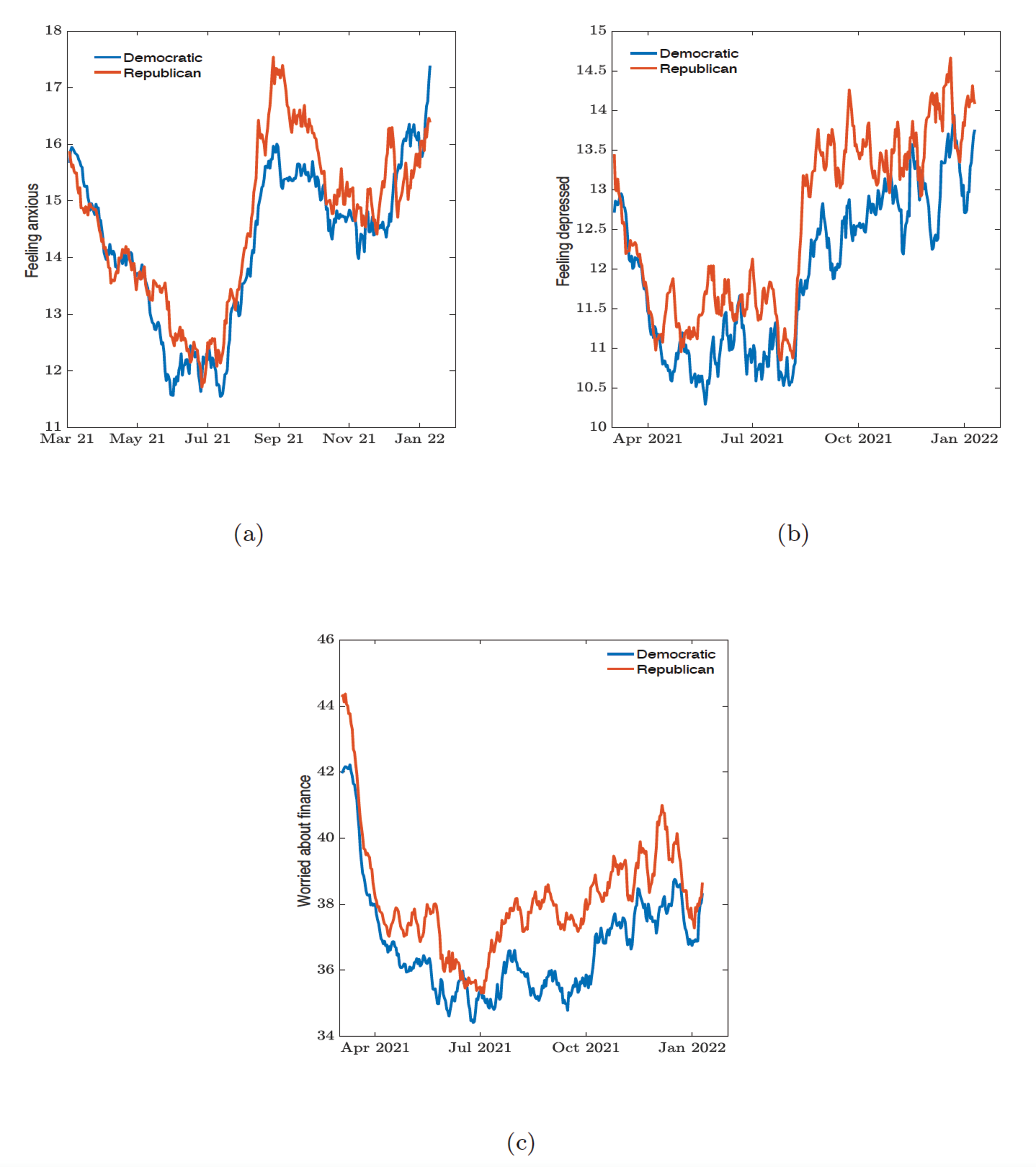

Supplement: S1 Fig — Percentage of individuals (a) feeling anxious, (b) feeling depressed, and (c) worried about finances. (TIF) [file pone.0286857.s001.tif]

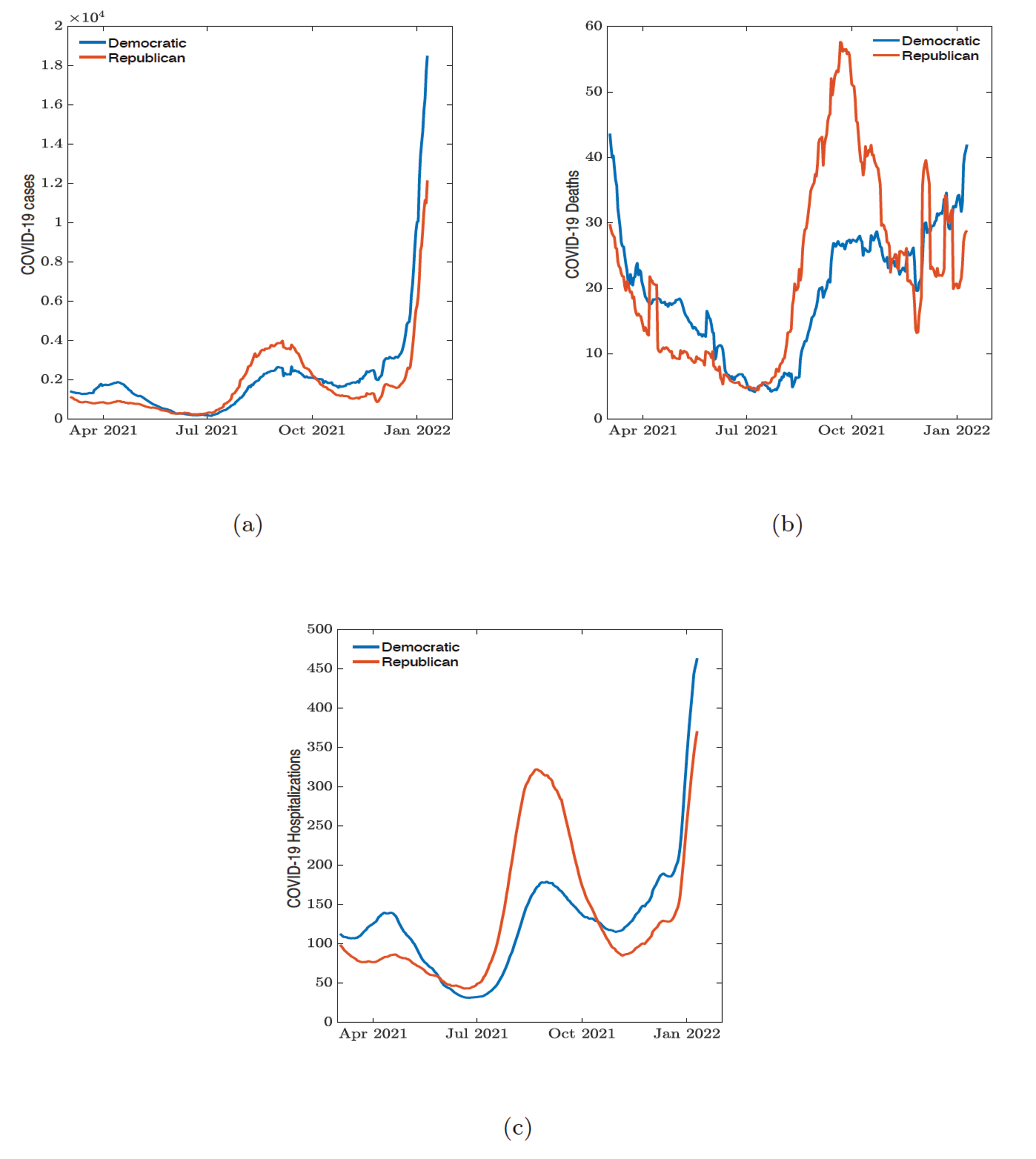

Supplement: S2 Fig — Daily COVID-19 (a) cases, (b) reported deaths, and (c) hospitalization cases. (TIF) [file pone.0286857.s002.tif]
