# Supplementary material for: Understanding mental health trends during COVID-19 pandemic in the United States using network analysis
Source: PLoS One. 2023 Jun 8;18(6):e0286857. doi: 10.1371/journal.pone.0286857 (PMC10249855; doi:10.1371/journal.pone.0286857)
Supplement: Attachment — Submitted filename: Response to reviewers comments.pdf [file pone.0286857.s003.pdf]

We would like to thank the reviewers for their insightful comment. We have revised the manuscript accordingly and describe the changes in detail below in italics highlighted in blue.

EDITOR:
1. Please ensure that your manuscript meets PLOS ONE's style requirements, including those for file naming. The PLOS ONE style templates can be found at
https://journals.plos.org/plosone/s/file?id=wjVg/PLOSOne_formatting_sample_main_body.pdf and
https://journals.plos.org/plosone/s/file?id=ba62/PLOSOne_formatting_sample_title_authors_affiliations.pdf

Response: *We have formatted the manuscript to align with the PLOS ONE style requirements as outlined in the links provided.*

2. Please update your submission to use the PLOS LaTeX template. The template and more information on our requirements for LaTeX submissions can be found at http://journals.plos.org/plosone/s/latex.

Response: *We have downloaded the LaTeX template to correctly format our manuscript, and have reviewed the submission guidelines.*

3. Thank you for stating the following in the Acknowledgments Section of your manuscript:
This research was funded by the National Science Foundation, grant number DMS2028297 and DMS2230117.

However, funding information should not appear in the Acknowledgments section or other areas of your manuscript. We will only publish funding information present in the Funding Statement section of the online submission form.
Please remove any funding-related text from the manuscript and let us know how you would like to update your Funding Statement. Currently, your Funding Statement reads as follows:

This research was funded by the National Science Foundation, grant number DMS2028297 and DMS2230117.The funders had no role in study design, data collection and analysis, decision to publish, or preparation of the manuscript.

Please include your amended statements within your cover letter; we will change the online submission form on your behalf.

Response: *We have removed statements related to funding from the Acknowledgement Section and other areas of the manuscript. We have also updated our cover letter to reflect the amended Funding Statement.*

4. We note that Figures 2, 4 and 5 in your submission contain [map/satellite] images which may be copyrighted. All PLOS content is published under the Creative Commons Attribution License (CC BY 4.0), which means that the manuscript, images, and Supporting Information files will be freely available online, and any third party is permitted to access, download, copy, distribute, and use these materials in any way, even commercially, with proper attribution. For these reasons, we cannot publish previously copyrighted maps or satellite images created using proprietary data, such as Google software (Google Maps, Street View, and Earth). For more information, see our copyright guidelines: http://journals.plos.org/plosone/s/licenses-and-copyright.

We require you to either (1) present written permission from the copyright holder to publish these figures specifically under the CC BY 4.0 license, or (2) remove the figures from your submission:

a. You may seek permission from the original copyright holder of Figures 2, 4 and 5 to publish the content specifically under the CC BY 4.0 license.

We recommend that you contact the original copyright holder with the Content Permission Form (http://journals.plos.org/plosone/s/file?id=7c09/content-permission-form.pdf) and the following text:
"I request permission for the open-access journal PLOS ONE to publish XXX under the Creative Commons Attribution License (CCAL) CC BY 4.0 (http://creativecommons.org/licenses/by/4.0/). Please be aware that this license allows unrestricted use and distribution, even commercially, by third parties. Please reply and provide explicit written permission to publish XXX under a CC BY license and complete the attached form."

Please upload the completed Content Permission Form or other proof of granted permissions as an "Other" file with your submission.

In the figure caption of the copyrighted figure, please include the following text: "Reprinted from [ref] under a CC BY license, with permission from [name of publisher], original copyright [original copyright year]."

b. If you are unable to obtain permission from the original copyright holder to publish these figures under the CC BY 4.0 license or if the copyright holder's requirements are incompatible with the CC BY 4.0 license, please either i) remove the figure or ii) supply a replacement figure that complies with the CC BY 4.0 license. Please check copyright information on all replacement figures and update the figure caption with source information. If applicable, please specify in the figure caption text when a figure is similar but not identical to the original image and is therefore for illustrative purposes only.
The following resources for replacing copyrighted map figures may be helpful:

USGS National Map Viewer (public domain): http://viewer.nationalmap.gov/viewer/

The Gateway to Astronaut Photography of Earth (public domain):
http://eol.jsc.nasa.gov/sseop/clickmap/

Maps at the CIA (public domain):

https://www.cia.gov/library/publications/the-world-factbook/index.html and

https://www.cia.gov/library/publications/cia-maps-publications/index.html

NASA Earth Observatory (public domain): http://earthobservatory.nasa.gov/

Landsat: http://landsat.visibleearth.nasa.gov/

USGS EROS (Earth Resources Observatory and Science (EROS) Center) (public domain):
http://eros.usgs.gov/#

Natural Earth (public domain): http://www.naturalearthdata.com/

Response: *The maps of the United States as used in Figures 2, 4, and 5 in the manuscript were generated by the authors using the R package usmap https://cran.r-project.org/web/packages/usmap/index.html). This package is an open source code developed by Paolo Di Lorenzo, and is under a GNU General Public License (Version 3). Since the communities were created based on our own data, we have not requested permission to use, nor removed the figures. We have amended our captions of these three figures to include the source information.*

5. Please review your reference list to ensure that it is complete and correct. If you have cited papers that have been retracted, please include the rationale for doing so in the manuscript text, or remove these references and replace them with relevant current references. Any changes to the reference list should be mentioned in the rebuttal letter that accompanies your revised manuscript. If you need to cite a retracted article, indicate the article's retracted status in the References list and also include a citation and full reference for the retraction notice.

Response: *We have reviewed the reference list to reflect the papers cited in our manuscript and there are no changes to our list.*

6. This manuscript has an essential theme. I liked reading it. Make changes to the description of the methodology and results so that these sections are easy to understand for readers who need to gain expertise in the methods used.

Response: *We have rearranged and re-worded the methodology, results, and discussion section so readers can better understand and follow the contents of the manuscript.*

Reviewer #1: Dear authors,

My congratulations for the quality of the manuscript and political and psychosocial approach to the impact of COVID-19 on the American population. It is certainly a type of study to be replicated in other countries.

Response: *Thank you for the kind comment and recommendation.*

REVIEWER #2:

This is a very relevant paper with a very important thematic. I liked reading it. The text flows very nicely, and the relevance of the study is emphasized throughout the paper. One thing that makes me feel uncomfortable overall is that I am not a specialist on the methods.
Response: *Thank you for the comment.*

The interpretations are hard, and the methodological discussion too. For the readers of Plos One, an interdisciplinary journal, it may be an obstacle for the reader. The interpretation of the results is compromised because of its difficulty, as it follows from the methodology section. Nevertheless, I leave it to the editor for a consideration, giving the preference of current readers.
Response: *We have reordered the manuscript and reworded the text from the methodology section to the discussion section to make it a lot easier to follow and understand.*

Bearing my limitations in mind, I have a few considerations:

define "connectome" in the abstract
define "connectome" for those not familiar with the area of study in the very first appearance on the paper.
Response: *We have given a brief definition of "connectome" in the abstract and given a detailed description in the Introduction section of the manuscript with references.*

dessazonalize all data for the analysis - sazonality is a challenge for quantitative methods. It usually follows as a standard procedure to dessazonalize data in order to have the meaningful curvatures in the analysis.
Response: *The regarding deseasonalization, we believe that deseasonalizing our data would not be necessary for the following reasons. The timeframe of our data is under a full year, making it difficult to determine any seasonal signal if any. While it may be arguable that there are seasonal fluctuations in individuals perceived mental health state, we would require long-term data to calculate the seasonal component, which is difficult given the length of the survey data we are using. We believe that the main driving factor for the survey results are based on COVID-19 cases, deaths, and hospitalizations.*

list on page 7 could be translated into a box, according to "Policy guideline that affects finances" and "Policy guideline that affects feeling anxious and depressed"

Response: *The policy guideline list in the Methods section has been converted into tables as suggested, where readers can distinguish between policies that affect finances, and policies that affect feelings of anxiety and depression.*

Figure 4: Country-wide maps grouping states with similar trends between 3/2/2021 to 1/10/2022 for (a) feeling anxious, (b) feeling depressed and (c) worried about finances indicators. The states with light blue, blue, and dark blue are in the same cluster. --> so that means that all country is in the same cluster? I could find out on the subtitle for figure 5 that my comment below its not the case. So please change figure's 4 subtitle.

Response: *We have fixed the caption label for Figure 4 to reflect the three separate clusters.*
